# Half-Octave Shift in Mammalian Hearing Is an Epiphenomenon of the Cochlear Amplifier

**DOI:** 10.1371/journal.pone.0045640

**Published:** 2012-09-25

**Authors:** Sripriya Ramamoorthy, Alfred L. Nuttall

**Affiliations:** 1 Oregon Hearing Research Center, Department of Otolaryngology, Oregon Health & Science University, Portland, Oregon, United States of America; 2 Kresge Hearing Research Institute, University of Michigan, Ann Arbor, Michigan, United States of America; University of Salamanca- Institute for Neuroscience of Castille and Leon and Medical School, Spain

## Abstract

The cochlear amplifier is a hypothesized positive feedback process responsible for our exquisite hearing sensitivity. Experimental evidence for or against the positive feedback hypothesis is still lacking. Here we apply linear control theory to determine the open-loop gain and the closed-loop sensitivity of the cochlear amplifier from available measurements of basilar membrane vibration in sensitive mammalian cochleae. We show that the frequency of peak closed-loop sensitivity is independent of the stimulus level and close to the characteristic frequency. This implies that the half-octave shift in mammalian hearing is an epiphenomenon of the cochlear amplifier. The open-loop gain is consistent with positive feedback and suggests that the high-frequency cut-off of the outer hair cell transmembrane potential in vivo may be necessary for cochlear amplification.

## Introduction

Our ability to hear low-level sounds is due to an amplification mechanism called the “cochlear amplifier” [1] in the inner ear. In acutely traumatized mammalian cochleae, maximum loss in auditory sensitivity does not occur at the same frequency as the sound-exposure, but approximately half octave higher [2]. This ‘half-octave shift’ is seen at the psychophysical, whole nerve, and indirectly at the single neuron level. Based on their experiments Cody and Johnstone deduced that basilar membrane (BM) nonlinearities would be the likely source of the half-octave shift. Indeed, direct experiments subsequently found that the BM motion [3], [4], [5] exhibits the half-octave shift as the intensity of the sound stimulus increases from about 20 dB SPL to above 100 dB SPL. Cochlear models [6], [7], [8] use the half-octave shift as an important “goodness” score to compare with measured data. The physiological origin of this frequency-shift, other than the fact that it is known to arise only when the cochlear amplifier is functional, is not completely understood. The cochlear amplifier is hypothesized to be positive feedback [1], but evidence for this hypothesis is still lacking. Mountain et al. [9], Mountain and Hubbard [10], and Nakajima et al. [11] demonstrated a model of the cochlear amplifier that is negative feedback at frequencies below the characteristic frequency (CF) and is positive feedback at frequencies near the CF. Elliott et al. [12] developed a state-space nonlinear feedback time-domain model which can potentially be used to investigate the nonlinear dynamics of the cochlear amplifier. Lu et al. [13] have suggested that the cochlear amplifier could be negative feedback even near the CF but still create amplification.

In this article, we use a linear frequency-domain model [14] of the cochlear feedback loop to deduce the closed-loop sensitivity and open-loop gain of the cochlear amplifier from diverse published measurements of BM velocity in sensitive mammalian cochlea, including our earlier measurement [4] and model [8]. These attributes of the cochlear amplifier were reviewed in Robles and Ruggero [15]. However, estimates and insights on the open-loop gain and closed-loop sensitivity are still lacking. We demonstrate that the frequency of peak closed-loop sensitivity is close to the CF and independent of the stimulus level. This shows that the half-octave shift, although occurring only when the amplifier is functional, is not a direct attribute of the cochlear amplifier. Second, we derive the complex open-loop gain of the cochlear amplifier from the complex closed-loop sensitivity for varying stimulus levels and show evidence to support the positive feedback hypothesis. The derived open-loop gain vs. stimulus level is used to shed light on the active process, and also leads us to propose that the high frequency cut-off of the outer hair cell (OHC) transmembrane potential is necessary (and is not a hindrance) for cochlear amplification.

## Methods

To derive the closed-loop sensitivity and open-loop gain of the cochlear amplifier from the measurements of BM vibration in sensitive mammalian cochlea the following assumptions are made about the feedback loop: (1) the active response of the cochlea to varying stimulus levels is considered to be quasi-linear following de Boer’s EQ-NL theorem [3]. (2) Strictly speaking, the active amplification in the cochlea could involve distributed feedback mediated by the active traveling wave propagating along the tonotopic axis from base to apex. However, past studies have indicated that the primary region of amplification is sufficiently localized to within a few hundred microns [16] or up to a millimeter [17]. In the basal turn guinea pig cochlea, one wavelength near the characteristic place is about few hundred microns. (the wavelength at best place is 200 µm at 16 kHz place in gerbils [18] and 500 µm at 12 kHz place in chinchilla [19]). These past studies are used as the basis to simplify the cochlear amplifier as a local feedback system. (3) The BM is considered as the feedback sensor here because extensive measurements of the BM vibration in sensitive mammalian cochlea are available from many laboratories including ours. However, these results need to be revised in the future as detailed measurements of the organ of Corti (OoC) vibration emerge [20]. The anticipated consequence of the use of BM as the feedback sensor is discussed in the Discussion section.

**Figure 1:**
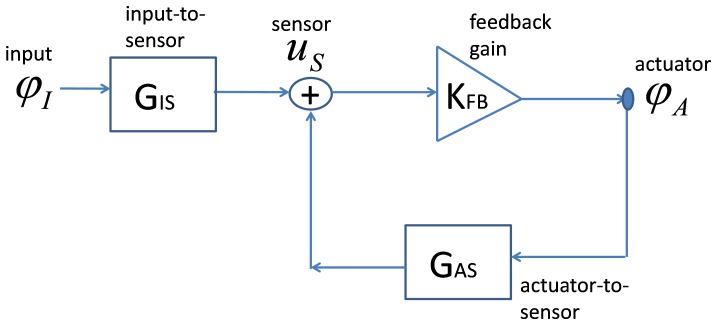
System block diagram for the linear local feedback model of the cochlear amplifier; G_AS_ is the transfer function from actuator to sensor, K_FB_ is the feedback gain, and G_IS_ corresponds to the passive transfer function from input to sensor. For other details, see text.

The closed-loop sensitivity can be derived as a function of the open-loop gain as follows (see **Fig. 1**). In **Fig. 1**, the input signal φ_I_ could be considered as the pressure at the ear canal or the displacement of the stapes. The term G_IS_ is the passive transfer function from the input to the sensor, φ_A_ is the actuator signal (such as the active force), G_AS_ is the transfer function from the actuator to the sensor, and K_FB_ is the feedback gain.

The passive displacement at the sensor is given by
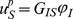
(1)


The active displacement at the sensor, which is for the feedback loop closed, is given by
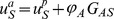
(2)


The actuator signal is 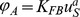.

The active and passive displacements at the sensor are therefore related as:
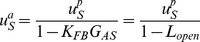
(3)Here, *L_open_* is the open-loop gain given by the product of the transfer functions in the feedback path. In other words, 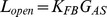. The ratio of active to passive displacements gives the closed-loop sensitivity of the cochlear amplifier. The closed-loop sensitivity and the open-loop gain are therefore related as (similar to common practice [21]):



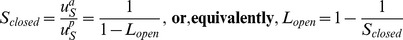
(4)If negative feedback is assumed to start-with, then the denominator for *S_closed_* in Eq. (4) will be ‘1+ *L_open_*’ which will change the phase of the open-loop gain determined from *S_closed_* by 180°. However, this representation does not affect the analysis. Also, replacing the displacement sensor by a velocity sensor in the above equations (equations 1–[Disp-formula pone.0045640.e002] [Disp-formula pone.0045640.e004] 4) does not change the open and closed-loop gain.

## Results

### Closed-loop Sensitivity of the Cochlear Amplifier

**Figure 2:**
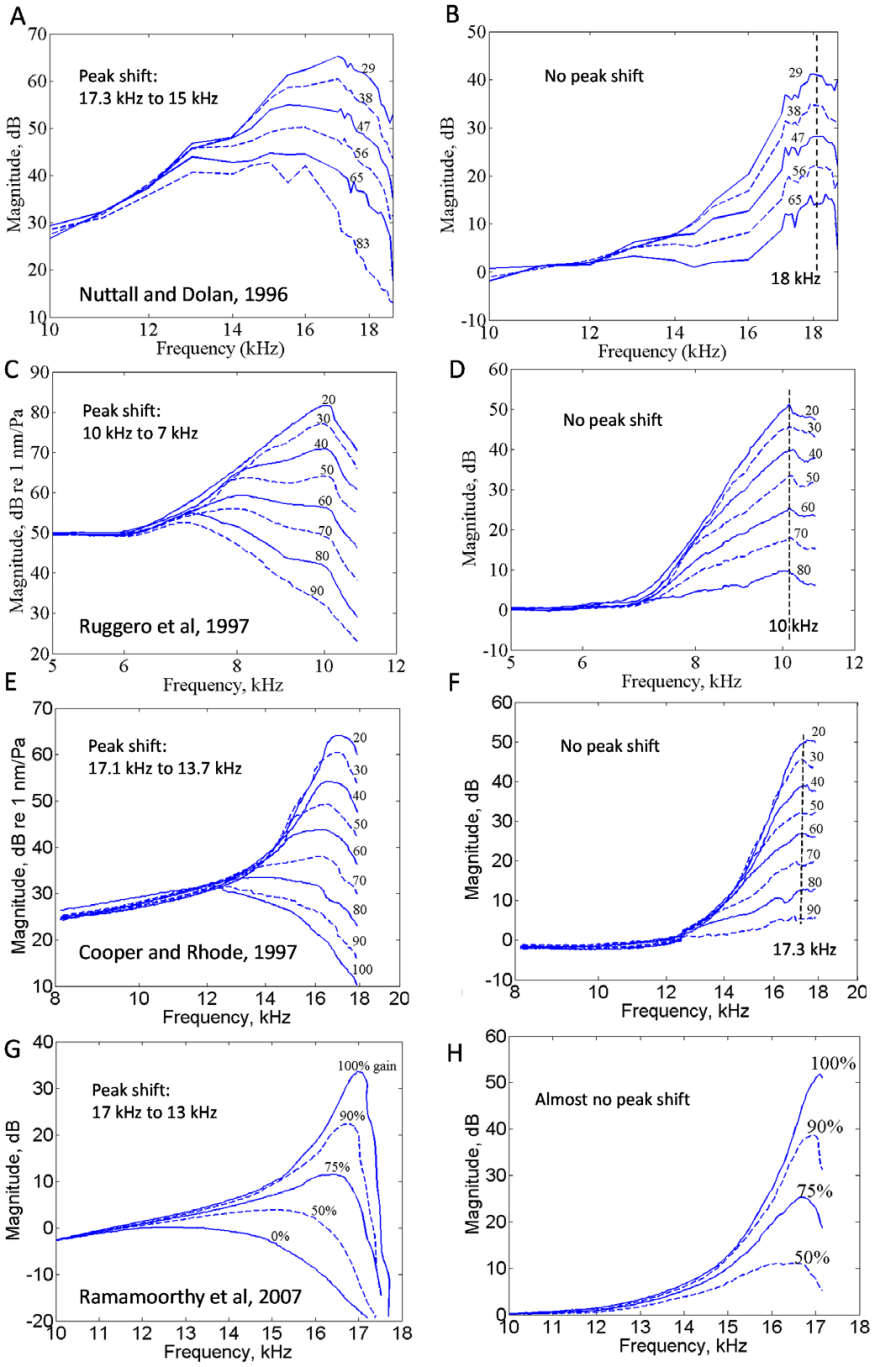
BM tuning curves for varying sound stimulus levels (left) and the corresponding closed-loop sensitivity derived from those measurements (right) in the basal turn mammalian cochlea. The left panel (**A**) is BM displacement relative to stapes from Nuttall and Dolan [4] in guinea pigs; (**C**) is BM displacement relative to pressure at ear canal in chinchilla from Rugerro et al. [5]; (**E**) is BM displacement re pressure from Cooper and Rhode [23] also in chinchilla; and (**G**) is BM re stapes from the model predictions in Ramamoorthy et al. [8]. The right panels (**B**), (**D**), (**F**), and (**H**) show the corresponding closed-loop sensitivities. In (**A**)–(**F**), the numbers on the plot indicate the stimulus level in dBSPL; in (**G**) and (**H**), the numbers represent percentage of maximum MET conductance slope vs. HB displacement used in the model. From all four datasets, the BM tuning curves demonstrate shift in peak frequency (half-octave shift) with changes in stimulus level, whereas the closed-loop sensitivities do not.

As is well known, the measured BM vibrations relative to acoustic stimulus demonstrate a downward shift in the frequency of the peak as the stimulus level is increased [15]. This shift in the frequency of peak BM displacement with stimulus level is the basis for the ‘half-octave shift’ observed in behavioral [2] and neural studies [22]. In the guinea pig data from [4] shown in **Fig. 2A**, the frequency of the peak BM velocity relative to stapes shifts from 17.3 kHz to 15 kHz for stimulus level changing from 29 dB SPL to 83 dB SPL. For the chinchilla data from [5] shown in **Fig. 2C**, it shifts from 10 kHz to 7 kHz for stimulus level change from 20 to 90 dB SPL. For the chinchilla data from [23], the frequency of the peak shifts from 17.1 kHz to 13.7 kHz (**Fig. 2E**) as the stimulus level changes from 20 to 100 dB SPL. Their respective closed-loop sensitivities can be determined following Eq. (4) as:
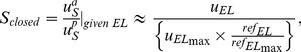
(5)assuming the BM vibration at the stimulus excitation level *EL_max_* (and above) is a good approximation for the passive or postmortem response. Ideally, *EL_max_* would be above 120 dB SPL, but this is not available for most published experimental data. *EL_max_* is 100 dB SPL for the Cooper and Rhode [23] data, 90 dB SPL for the Ruggero et al. [5] data, and 83 dB SL for the Nuttall and Dolan [4] data. The subscript ‘EL’ refers to the sound stimulus excitation levels starting from about 20 or 30 dB SPL (see **Fig. 2** legend). Here, 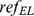 refers to the reference used in the experiment at the same stimulus level as used for BM vibration recording. In the data considered here, the reference is either acoustic pressure at the ear-canal or stapes vibration.

The closed-loop sensitivity derived using Eq. (5) is shown in **Fig. 2B** for the Nuttall and Dolan [4] data, **Fig. 2D** for the Ruggero et al. [5] data, and **Fig. 2F** for the Cooper and Rhode [23] data. These plots show that, unlike the BM tuning in **Fig. 2A**, **Fig. 2C**, and **Fig. 2E**, the closed-loop sensitivity does not demonstrate shift in peak frequency as the stimulus level changes. The frequency of peak sensitivity is at or very near the characteristic frequency for the measured tonotopic location and, more importantly, this peak does not shift with stimulus intensity. Only the level of closed-loop sensitivity increases with decrease in the stimulus intensity.

Similar behavior is also seen in the response predicted by the mechano-electrical-acoustic finite element model of the cochlea [8]. The BM velocity relative to the stapes (**Fig. 2G**) shows the peak frequency decreasing from 17 kHz to 13 kHz with increase in stimulus level, which is represented as decreasing gain. The frequency of peak closed-loop sensitivity, on the other hand, is nearly constant at 17 kHz with changes in stimulus intensity.

**Figure 3:**
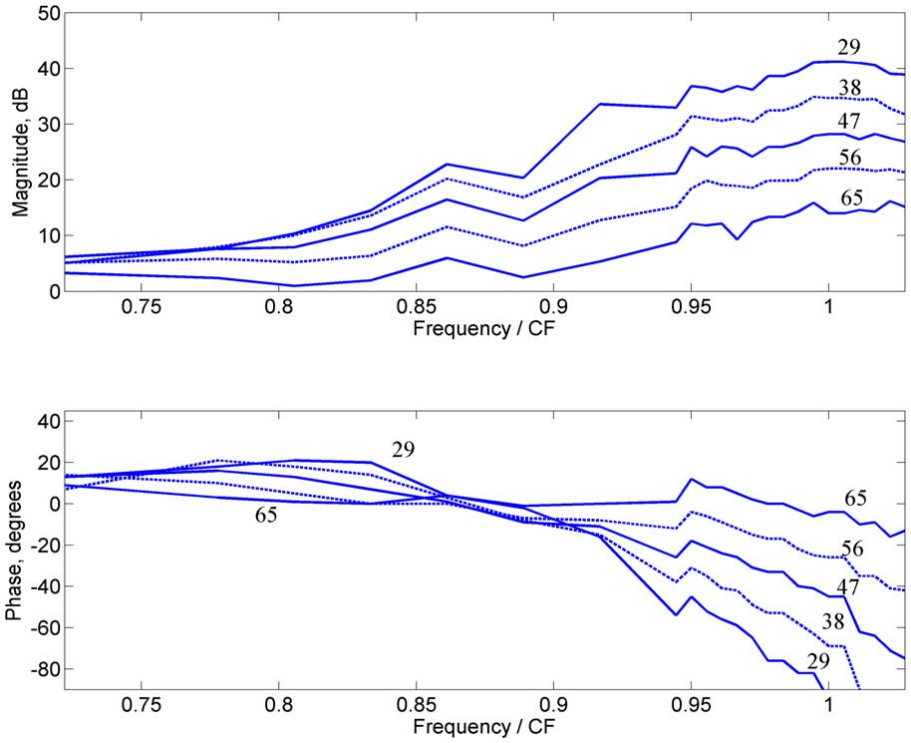
This figure shows the complex closed-loop sensitivity from guinea pig 2381-2SE [4] for varying stimulus levels. The phase (bottom panel) demonstrates lead below about 16 kHz and lag above 16 kHz. The CF is 18 kHz for this data. The numbers on the plot indicate the stimulus level.

**Figure 3** shows the complex closed-loop sensitivity derived from Nuttall and Dolan [4]. The magnitude (top panel) is same **Fig. 2B**. The phase for closed-loop sensitivity (bottom panel), also given in Fig. 8 of Robles and Ruggero [15], leads below 16 kHz and lags above 16 kHz (close to CF) for the closed-loop sensitivity. This phase is similar to that of a stable closed-loop pole near the CF. This is also a common finding among auditory nerve fibers [22]. That the zero-phase is not at the CF as discussed in Robles and Ruggero [15], but at a lower frequency, suggests that the feedback sensor might be another structure in the OoC (see Discussion section).

### Open-loop Gain of the Cochlear Amplifier

**Figure 4:**
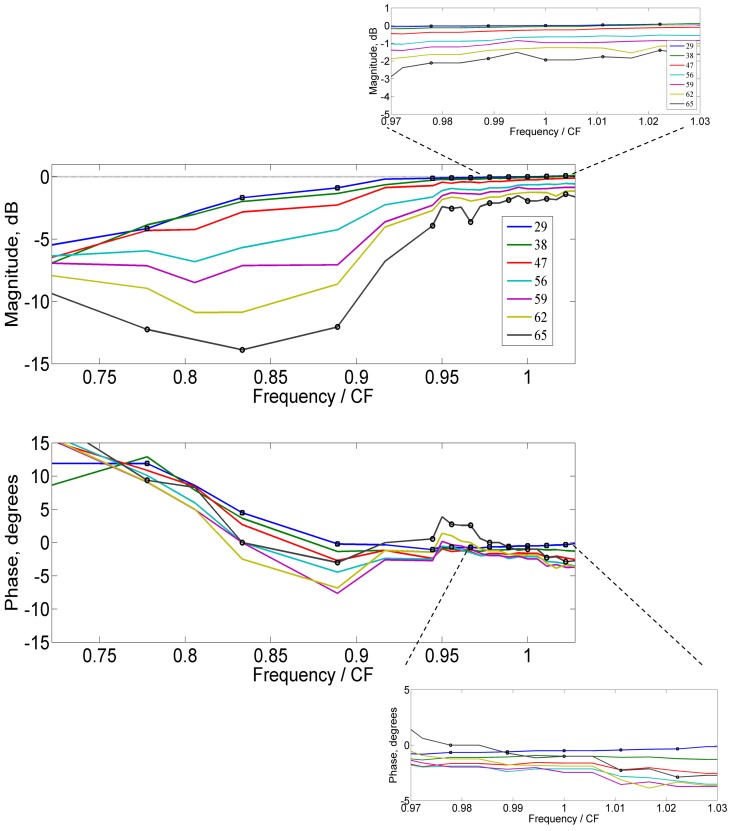
The open-loop gain vs. non-dimensional stimulus frequency for varying stimulus levels (numbers in dBSPL) derived from the BM measurements for guinea pig (2381-2SE) from [4]: magnitude (top) and phase (bottom). The CF is 18 kHz for this data. The frequency range is 13 kHz to 18.5 kHz; insets zoom into 17.5 kHz to 18.5 kHz.

The complex open-loop gain for varying stimulus levels is derived from the complex closed-loop sensitivity using Eq. (4) for BM vibration from [4]. **Figure 4** shows the magnitude in dB (top panel) and the phase in degrees (bottom panel). For the lowest stimulus level, the open-loop gain *L_open_* is close to unit magnitude and zero phase over a limited bandwidth around the CF. The result indicates that the cochlear amplifier operates in positive feedback. If the amplifier were negative feedback, *L_open_* would have phase close to 180° (for the sign convention used in Eq. (4)). The closed-loop sensitivity is highest at the frequency where the open-loop gain is close to 1. At low frequencies, even at the lowest stimulus level, the open-loop gain is less than 1 and has non-zero phase.

As the stimulus level increases the magnitude of the open-loop gain decreases as shown in **Fig. 4** (top panel). The insets zoom into the frequencies around the CF. The decrease in open-loop gain as the stimulus level is increased from 29 dB SPL to 65 dB SPL is only a factor of 0.75 (or 2.5 dB reduction). Furthermore, the phases of the open-loop gain change less than 5° with stimulus level near the CF (**Fig. 4**, bottom panel). In other words a 2.5 dB decrease in the open-loop gain magnitude and less than 5° change in phase leads to a reduction of nearly 27 dB in the closed-loop sensitivity (**Fig. 2B**). The small reduction in the magnitude of the open-loop gain with increase in stimulus level appears to be consistent with a similar decrease in the slope of mechano-electrical transduction (MET) conductance vs. HB displacement. In the model [8] shown in **Fig. 2H**, 75% (or 2.5 dB) reduction in MET conductance slope vs. HB displacement leads to a reduction in the closed-loop sensitivity of about 27 dB. It is interesting to note here that a similar quantitative relationship between the OHC receptor current and elevation of neural threshold was suggested in Fig. 11 of Patuzzi et al [24]. Furthermore, the small phase of the open-loop gain suggests that the cochlear amplifier, by itself, does not add significant non-minimum-phase delay.

## Discussion

In this article, linear control theory is applied to derive the closed-loop sensitivity and open-loop gain of the cochlear amplifier using the simplifying assumptions of quasi-linear behavior and local feedback. The implications of the results for cochlear mechanics are discussed below.

### (1) Half-octave Shift is an Epiphenomenon of the Cochlear Amplifier

We show that the closed-loop sensitivity (**Fig. 2B, 2D, 2F, and 2H**), which removes the passive response, and represents the characteristic behavior of the cochlear amplifier by itself, does not exhibit a shift in the frequency of peak sensitivity with change in sound stimulus level. This result demonstrates that the half-octave shift is an epiphenomenon of the cochlear amplifier. The shift seen is due to a combination of the negatively sloped asymmetric tail of the passive BM response (at frequencies higher than the passive best frequency) along with the changes in the level of the closed-loop sensitivity.

**Figure 5:**
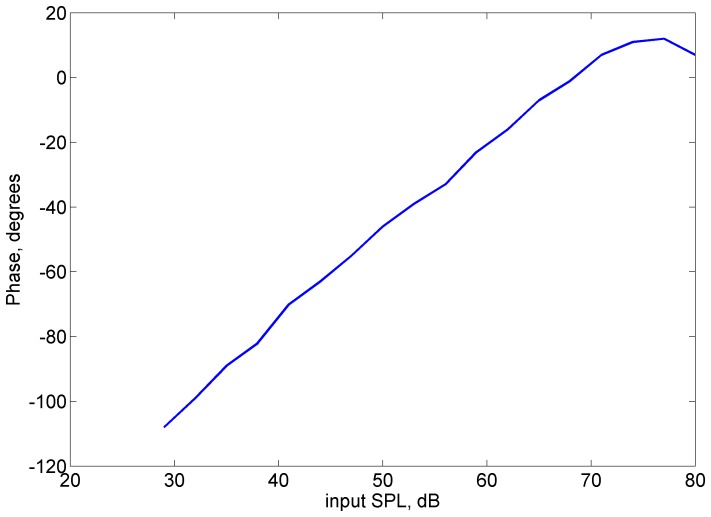
Phase of closed-loop sensitivity at 18 kHz CF vs. stimulus level for guinea pig 2381-2SE from [4].

### (2) The Frequency of Peak Closed-loop Sensitivity is Independent of Stimulus Level

The frequency of peak closed-loop sensitivity at a given tonotopic location does not shift as the stimulus level changes, and this frequency is very close to the CF. This peak-frequency therefore appears to be a characteristic of the organ of Corti. It could be indicative of a second resonance in the organ of Corti [25], [26]. A conceivably related phenomenon is the appearance of peaks at two frequencies in the BM response to electrical current injected into guinea pig cochlea [27], [28]: one at the passive best frequency and other at the CF.

### (3) Anticipated Effect of the Use of BM as the Feedback Sensor

A mechanical network model of the organ of Corti, such as used in [29], could be utilized to estimate the likely effect of the use of BM as the feedback sensor to determine the closed-loop sensitivity. From Eq. (12) in that article, the ratio of RL to BM displacements – in response to only the somatic force – is given by the negative of the ratio of BM mechanical impedance to RL-tectorial membrane (TM) impedance. The complex-valued ratio of RL to BM displacement evaluated at 19 kHz CF/3 mm tonotopic location using parameters from that article, although about −5 at low frequencies (see the experimental data from [30]), changes to about 2j near the CF owing to inertia of the organ of Corti structures. This 90° phase lead of RL relative to BM around the CF appears consistent with the experimental data for lowest stimulus level in Fig. 5a of [20]. As the stimulus level increases, this phase difference is expected to progressively decrease due to the reduced influence of the active (somatic) force on the organ of Corti vibration. The closed-loop phase at the CF (18 kHz) as a function of stimulus level from the bottom panel of **Fig. 3** for the guinea pig 2381-2SE is shown in **Fig. 5**. This phase resembles the lag in BM response relative to RL in a sensitive cochlea expected based on the analysis discussed above, as well as based on Fig. 5a of [20]. This result suggests that if the RL were chosen as the feedback sensor instead of the BM, the closed-loop sensitivity would cross zero phases at the CF. Further experiments are necessary to confirm whether the RL is the feedback sensor.

### (4) High-frequency Cut-off of the OHC Transmembrane Potential may be Necessary

The literature in the field of cochlear mechanics has widely questioned the efficacy of the OHC somatic electromotility process by virtue of the high-frequency cut-off of its transmembrane potential (see [31] for a review). We recently demonstrated [29] that the (cut-off) value of the OHC transmembrane potential *in vivo* is sufficient to cause expected power amplification in the high-frequency basal region in mammalian cochlea. The open-loop gain derived in this article further extends this concept and indicates that the cut-off may be a necessary (not just sufficient) condition. If the OHC transmembrane potential did not cut-off at high frequencies and were suppose, 10 times higher, then the open-loop gain would have a magnitude close to 10 near the CF instead of 1. For open-loop gain magnitude close to 10 (and nearly independent of the open-loop gain phase), from Eq. (4), the closed-loop sensitivity would be much smaller (≈ −20 dB) at the CF. Note that this simple illustration considers a change only in the magnitude of the open-loop gain. In general, both magnitude and phase are important for amplification and stability in closed-loop. Thus, the near-unity open-loop gain derived from *in vivo* measurements of the BM vibration near the CF suggests that the high-frequency cut-off of the OHC transmembrane potential may be necessary for cochlear amplification. This hypothesis remains to be verified with direct experiments.
